# Response mechanism to heat and hypoxia stress in hard clam: insights from antioxidation and lipid metabolism

**DOI:** 10.1186/s12983-026-00622-5

**Published:** 2026-06-30

**Authors:** Zhi Hu, Mei-Jie Yang, Xing-Hao Ma, Pu Shi, Tao Zhang, Hao Song

**Affiliations:** 1 Key Laboratory of Mariculture of Ministry of Education, Ocean University of China, Qingdao, 266003 P. R. China; 2 CAS Key Laboratory of Marine Ecology and Environmental Sciences, Institute of Oceanology, Chinese Academy of Sciences, Qingdao, 266071 China; 3 Center for Ocean Mega-Science, Chinese Academy of Sciences, Qingdao, 266071 China; 4 University of Chinese Academy of Sciences, Beijing, 100049 China; 5 Shouguang Marine Fishery Development Center, Weifang, 262700 China; 6 School of Marine Sciences, Ningbo University, Ningbo, 315211 China

**Keywords:** Antioxidant response, Heat, Hypoxia, Lipidomics, *Mercenaria mercenaria*

## Abstract

**Background:**

The hard clam (*Mercenaria mercenaria*) is native to the coast of the United States and Canada. Following its introduction to China, it has become an economically important bivalve species cultured in ponds. Extreme heat and hypoxia events severely impact the physiological status of marine organisms. Excess reactive oxygen species produced by environmental stress can lead to antioxidant stress response and lipid remodeling. Antioxidant stress response and lipid metabolism can reveal the response mechanism and adaptation ability of organisms to environmental changes.

**Results:**

In the present study, coupled lipidomic and biochemical approaches were employed to reveal the response mechanism to heat and hypoxia stress at the antioxidant stress and lipid metabolism level in the hard clam. The antioxidant system was enhanced showing stress-specific to increase total antioxidant capacity (T-AOC) capacity and maintain relatively stable malondialdehyde (MDA) level. 913 lipid metabolites were identified by widely targeted lipidomic analysis. Glycerophospholipids (GP), glycerolipids (GL), and sphingolipids (SP) were the main differential expression lipid metabolites. Lipid remodeling of the gill tissue was mainly caused by GP, GL and SP metabolism in the hard clam.

**Conclusion:**

Activation of the antioxidant system which evidenced by elevated T-AOC, can alleviate oxidative damage, thereby preventing excessive accumulation of MDA under environmental stress. Concurrently, increased abundances of GP and SP metabolites drive remodeling of membrane lipids, which is critical for maintaining membrane fluidity and structural integrity during stress. Furthermore, the accumulation of GL metabolites functions as thermos-protectant. This study expands our understanding of hard clam responses to heat and hypoxia stress at the antioxidant and lipid metabolism level.

**Supplementary information:**

The online version contains supplementary material available at https://doi.org/10.1186/s12983-026-00622-5.

## Background

Marine organisms are frequently faced various environmental stressors including heat, hypoxia, etc. Extreme heat and hypoxia stress are considered as important stressors to aquatic animals under global warming scenarios [1]. Rising temperatures lead to lower dissolved oxygen levels [2], and hypoxia may exacerbate under high temperatures [3]. Global climate change presents unprecedented challenges to aquaculture, primarily through the combined effects of high temperature and low dissolved oxygen [4]. Elevated temperatures and low oxygen content are the potential environmental stressors contributing to frequent mass mortality events in bivalves [5]. For example, it was reported that in the summer of 1998, heat and hypoxia were the main causes of mass mortality of *Crassostrea gigas* (Thunberg, 1793) in Puget Sound and Tomales Bay [6]. Coastal warming and associated hypoxia driven by climate change are increasingly affecting coastal and estuarine bivalve fisheries [7, 8]. Due to their limited mobility, most adult bivalves must adjust their physiological and biochemical responses to withstand environmental stress. Therefore, it is vitally important to investigate the molecular and physiological responses of bivalves to heat and hypoxic stress.

To date, research on marine bivalves has largely focused on regulatory mechanisms under single stressors, whereas the combined effects of heat and hypoxia remain comparatively underexplored. Under such combined stress, mitochondrial integrity and function can be injured [9]. Mitochondrial dynamics process (including fusion, fission, and mitophagy) plays a crucial role in maintaining mitochondrial homeostasis and function [10]. Organisms adjust their energy metabolism as a vital adaptive strategy to enhance stress tolerance [11], often marked by an enhancement of anaerobic metabolism [10, 12–14]. Transcriptomic and proteomic studies have provided comprehensive insights into heat and hypoxia adaptation in bivalves, revealing numerous stress-responsive genes and pathways. For instance, the great scallop *Pecten maximus* (Linnaeus,1758) appears to modulate the abundance of energy metabolism-related proteins to conserve energy, and apoptosis may further contribute to resistance under combined stress [13, 15]. Significantly enriched pathways under heat and hypoxia include protein processing in the endoplasmic reticulum and ubiquitin-mediated proteolysis in hard clam *Mercenaria mercenaria* (Linnaeus,1758) [16]. Additionally, the heat shock protein (HSP) and hypoxia-inducible factor 1 (HIF-1) signaling pathways are commonly activated in three scallops [17]. Studies have also elucidated the roles of key gene families, such as HSP70 and the MPAKK family, in these adaptive responses [18, 19]. Despite these advances, heat and hypoxia stresses could affect a series of physiological and biochemical responses. The overall adaptive mechanism is likely shaped by multiple interconnected processes. Several critical biological processes (such as antioxidant response) remain to be fully clarified, highlighting the need for further investigations.

Environmental stress can induce Reactive Oxygen Species (ROS) accumulation in bivalve [20]. Excess ROS are deleterious and toxic products, which can induce oxidative stress causing damage to intracellular proteins and organelles, lipid peroxidation, Deoxyribonucleic acid (DNA) fragmentation, apoptosis, etc [21]. The organisms have developed sophisticated antioxidant systems including antioxidant enzymes and nonenzymatic antioxidants to degrade ROS to substances with lower toxicity. Lei et al. (2016) noted that small molecular antioxidants constitute of the primary defense mechanism against ROS directly to prevent or eliminate oxidative stresses in mammals [22]. Glutathione (GSH) is a kind of predominant intracellular antioxidant, which act as ROS scavenger [21]. The elevated content of GSH could be found in bivalve under hypoxia or heat stress [23–26]. Under high temperature stress, hypotaurine content increased in a temperature-dependent manner in *Ostrea edulis* (Linnaeus, 1758), which involved in protection cells from oxidative damage [27]. Antioxidant enzymes detoxify ROS into less reactive species. Among the various enzymes involved in antioxidant defense, Superoxide Dismutase (SOD), Catalase (CAT), and Glutathione Peroxidase (GPx) are considered the most crucial in protecting organisms from oxidative stress [22]. SOD catalyze the conversion of O^2−^ into H_2_O_2_ [28], and then CAT or GPx catalyzes the decomposition of H_2_O_2_ into O_2_ and H_2_O [29, 30]. Moreover, Glutathione Reductase (GR) and Glutathione S-transferase (GST) play a role in GSH cycle and detoxification of reactive electrophilic compounds, respectively [21]. Numerous of publications have shown these antioxidant enzymes involve in maintain ROS homeostasis under heat or hypoxia in bivalves [20, 23–25, 31–35].

As mentioned above, excess ROS can induce oxidative stress causing damage to lipid, so the lipid metabolism should be not ignored. Lipids are ubiquitous in marine invertebrates, and are essential for modulating energy metabolism, facilitating cellular signaling, and preserving membrane integrity [36]. Lipid metabolism plays a key role in adaptation to environmental stress in marine bivalve. For example, elevated content of glycerophospholipids may help to maintain the structure and function of the membrane in razor clam *Sinonovacula constricta* (Lamarck, 1818) [37]. North oyster *C. gigas* enhances membrane fluidity adapt lower temperatures through higher unsaturated fatty acids and desaturation index than south oyster *C. angulata* (Lamarck, 1819) [38]. Sphingolipids and sterol lipids content may play a critical role in short-term temperature adaptation, while glycerophospholipid alterations may prioritize dynamic lipid modifications over abundance changes in oyster [39]. Alterations in lipid profiles provide insights into the underlying response mechanisms and adaptive capacity of organisms to environmental changes. Lipidome, originally a branch of “metabolome”, has become an independent field of study due to its structural and functional diversity and high endogenous abundance [40, 41]. It can conduct a comprehensive qualitative and quantitative analysis of lipids in organisms or cells, analyze the changes in their composition and content, screen out differential expression lipid metabolites to reveal the regulatory mechanisms at lipid metabolism level [42]. However, the application of lipidome in bivalve coping with environmental stress is relatively scarce [43]. The link of lipid metabolism and antioxidant responses should be also explored.

*Mercenaria mercenaria*, commonly known as the hard clam, is a valuable fishery resource in North America, with high economic and ecological importance [44]. After introduction, it has become a popular pond cultured bivalve along coastal of China [14, 45]. As a typical buried bivalve, the hard clams are frequently exposed to heat and hypoxia stress, especially in summer. In situ water temperature monitoring in Cedar Key (the main area of hard clam aquaculture in Florida) revealed that summer temperatures at hard clam grow-out sites can reach up to 35 °C [46, 47]. Moreover, the expected volume of near-anoxic water (DO < 0.2 mg/L) increased from zero in 1950 to 3.6 × 10^9^ m^3^ (95% CI: 2.4 × 10^9^ to 5 × 10^9^) in 2001 in Chesapeake Bay [48]. Previous study has shown that lipid metabolites (such as fatty acyl (FA), glycerolipids (GL), glycerophospholipids (GP)) account for nearly one-third of the total differentially expression metabolites, and some small molecule antioxidants including ascorbic acid, myoinositol involved in relieve ROS stress [14], indicating that lipid metabolism and antioxidant response are critical in enabling hard clams to adapt to heat and hypoxia stress. Therefore, the hard clam can be used as an ideal organism to study the lipid metabolism and antioxidation response for heat and hypoxia stress. We speculated that the antioxidant system was activated under heat and hypoxia stress showing stress-specific. Through enhancing antioxidant response and adjusting lipid metabolism to maintain or eliminate lipid peroxidation.

To address these gaps, in the present study, the antioxidant activity and non-enzymatic antioxidant content were measured to explore the antioxidant pattern and lipid peroxidation level. Furthermore, gill lipid metabolites were detected by widely targeted lipidomic technology, and then the differential expression lipid metabolites were analyzed. Moreover, the lipid metabolism genes expression patterns were further explored to reveal the molecular mechanism of lipid metabolism. This study expands our understanding of hard clam responses to heat and hypoxia stress at the antioxidant and lipid metabolism level.

## Materials and methods

### Experimental clams, stress challenge and sample collection

The healthy hard clams which shell was intact without any damage used in the present study were collected from the Binzhou culture ponds (Shandong Prince, China). The average shell length was 44.46 ± 4.83 mm and the average wet weight was 28.99 ± 8.85 g. The acclimation conditions were the water temperature was 18 – 20 °C, the salinity was ~30 psu and the dissolved oxygen (DO) was ＞6 mg/L. The acclimation period was last for two weeks. During acclimation period, the hard clams were fed by commercial *Spirulina* powder once a day. There was no mortality of the hard clams during the laboratory acclimation. The novel hypoxia simulation device designed by Li et al., (2019a) was employed to carry out the stress challenge experiment [49]. The details of the novel hypoxia simulation device could be found in Li et al., (2019a) [49]. Based on distribution area ecological data and a literature summary [10, 14, 16, 46, 47] of tolerance of adult clams, the heat and hypoxia stress were set 35 °C and 0.2 mg/L, respectively. One negative control group (NC, normal condition) and three stress groups including only heat stress (HT, 35 ℃); only hypoxia stress (LO, 0.2 mg/L); and heat combined with hypoxia stress (HL, 35 °C, 0.2 mg/L) were set up. In brief, there were four same environmental simulation systems in this device. A supply tank where to adjust of the DO concentration and water temperature was under four replicate experimental tanks. It contained a heater and sensors for control DO and temperature. Temperature adjustment was achieved by heater heating and DO adjustment was achieved by bubbling in nitrogen under the control of solenoid valves, respectively [49]. Once the experimental conditions of temperature or/and DO level was reached, the hard clams were abruptly exposed to the environmental simulation systems and remained in these experimental conditions. The temperature or/and DO value were recorded every one hour. Each groups contained 80 hard clams. Based on our previous study, all the hard clams in the NC group and LO group were found alive, showing 0% mortality after 2 days of the test duration. The clam mortality rate was 28.75% and 52.5% after 48 h stress in the HT group and HL group [14]. Based on a literature summary, plenty of studies used mortality approximately 50% as the sampling time point [14, 50–52]. So, we chose two days as the sampling time point. Gills were selected as the target tissue because they were the major respiratory organ in marine bivalves. Furthermore, gills were directly exposed to the fluctuations in the ambient oxygen levels hence sensitive to environmental stress [14, 16]. Although the function of tissues can be temporarily suppressed during passive survival, gill was key organ, whose function during environmental stress was maintained by complex and tightly regulated stress response mechanisms, including lipid metabolism and antioxidative defense mechanisms [39, 52].

### Antioxidant biochemical assays

Gill tissues were sampled from three individuals and pooled into one tube as a biological replicate. Each group contained six biological replicates. The activities of glucose-6-phosphate dehydrogenase (G6PDH) was determined using commercial kit (Quanzhou Ruixin Biological Technology Co., LTD, China) following the manufacturer’s protocol. The activities of enzymes including SOD, CAT, GR, GST and GPx, and the content of reduced GSH, Malondialdehyde (MDA) and Total Antioxidant Capacity (T-AOC) were determined using commercial kits (Nanjing Jiancheng Bioengineering Institute, China) following the manufacturer’s instructions.

The targeted metabolite analysis was carried out by Metware Biotechnology Co., Ltd. (Wuhan, China). Gill tissues were sampled from three individuals and pooled into one tube as a biological replicate. Each group contained three biological replicates. Appropriately 50 mg sample was mixed with 500 µL of 70% methanol and vortexed for 3 min, and then centrifuged (12000 rpm, 4°C) for 10 min. 300 μL supernatant was carefully taken by a pipetman (Eppendorf, Germany), and and placed in −20 °C for 30 min. The supernatant was centrifuged (12000 rpm, 4°C) for 10 min. 200 μL supernatant was transferred through Protein Precipitation Plate and then analyzed by an LC-ESI-MS/MS system. The details of system could be found in supplementary materials. Analyst 1.6.3 was used for data acquisitions. Multiquant 3.0.3 was used to quantify all targeted metabolites.

A nonparametric Mann-Whitney test was performed using SPSS 22.0 (SPSS Inc., Chicago, USA) to assess variations in antioxidant biochemical assays across the experimental and negative control groups. The significance level was set at *p* < 0.05.

### Lipidomic profiling

For lipidomic profiling, gill tissues were sampled from three individuals and pooled into one tube as a biological replicate. Each group contained a total of five biological replicates. All the lipid metabolite extraction, detection, identification, and quantification were performed by Wuhan MetWare Biotechnology Co., Ltd (Wuhan, Hunan province, China).

Lipid metabolites extraction was performed according to the standardized flow. In brief, 20 ± 2 mg sample of each biological replicate was thawed on ice. A ball mill (MM400, Retsch) was used for sample homogenize. The samples were homogenized (30 Hz) for 20 s with precooled steel balls and the centrifuged (3000 rpm, 4 °C) for 30 s. 1 mL extraction solvent (MTBE: MeOH = 3:1, v/v) containing internal standard mixture was added and then whirled for 15 min by VORTEX-5 (Kyllin-Bell, China). The internal standard mixture included LPC(16:0)-d31, Cer(d18:1/4:0), PC(13:0/13:0), DG(12:0/12:0/0:0) and TG(17:0/17:0/17:0) et al (supplementary materials). 200 μL water was added and whirled for 1 min, and then centrifuge for 10 min (12,000 rpm, 4 °C). 200 μL supernatant was carefully aspirated by a pipetman (Eppendorf, Germany), and then evaporated by a vacuum concentrator (CentriVap, LABCONCO). 200 μL mobile phase B was used for dry extract reconstituted and whirled for 3 min, and then centrifuged (12000 rpm, 4 °C) for 3 min. The supernatant was used for LC-MS/MS analysis.

The gill lipid metabolites were detected by Ultra Performance Liquid Chromatography UPLC (ExionLC AD，https://sciex.com.cn/) and Tandem mass spectrometry MS/MS (QTRAP®, https://sciex.com.cn/). The details of UPLC analytical conditions including column, solvent system, etc and MS condition including electrospray ionization ESI source operation parameters could be found in supplementary materials. The effluent was alternatively connected to an ESI- triple quadrupole-linear ion trap QTRAP-MS. The QTRAP® LC-MS/MS System which equipped with an ESI Turbo Ion-Spray interface was employed to LIT and triple quadrupole QQQ scan. The Analyst 1.6.3 software was used to operate in positive and negative ion mode. QQQ scans were employed to perform multiple reaction monitoring MRM experiments. The collision gas was set 5 psi.

### Lipidomic bioinformatic analysis

The Analyst 1.6.3 software was used for analyzing the mass spectrum data. The metware database MWDB database built by Metware Biotechnology Co., Ltd. was used for lipid metabolites detection. Principal Component Analysis (PCA) was performed by the prcomp function in R to determine the relationships of the identified metabolites among the tested samples. Furthermore, the Orthogonal Partial Least Squares Discriminant Analysis (OPLS-DA) was performed by R package MetaboAnalystR (v1.0.1). The differential expression lipid metabolites between groups were log_2_ and mean centered. Lipid metabolites with variable importance in projection (VIP) ≥1 and |Log_2_FC (fold change)| ≥1 were deemed differentially expressed [14]. The differential expression lipid metabolites were annotated by Kyoto Encyclopedia of Genes and Genomes KEGG compound database and then mapped. Pathways were analyzed using metabolite sets enrichment analysis (MSEA), with significance assessed via hypergeometric testing (*p* < 0.05).

### Lipid metabolism gene expression analysis

To reveal the lipid metabolism gene expression pattern among treatment groups and control group, we examined the transcriptomic profiles by using RNA-seq. Gill tissues were sampled from three individuals and pooled into one tube as a biological replicate. Each group contained three biological replicates. The details of libraries construction could be found in supplementary materials. The prepared libraries were sequenced on an Illumina NovaSeq 6000 platform by Majorbio Bio-pharm Biotechnology Co., Ltd (Shanghai, China). The 150 bp raw data was deposited in the NCBI SRA (PRJNA973806). Clean data were obtained by using fastp with default parameter. The hard clam reference genome [51] was selected as a reference. The filtered reads were mapped to the *M. mercenaria* genome by HISAT2. The readcounts (the numbers of reads mapped to each transcript) were quantified by using StringTie. The differential expression gene analysis was performed using the DESeq2. Genes with |log_2_(foldchange)| ≥1 and P-adjust ≤ 0.05 were deemed differentially expressed. To reveal the lipid metabolism gene expression pattern, the *M. mercenaria* genome proteins were annotation by using KEGG.

## Results

### Effects of heat and hypoxia stress on variation of antioxidant related biochemical assays

**Fig. 1:**
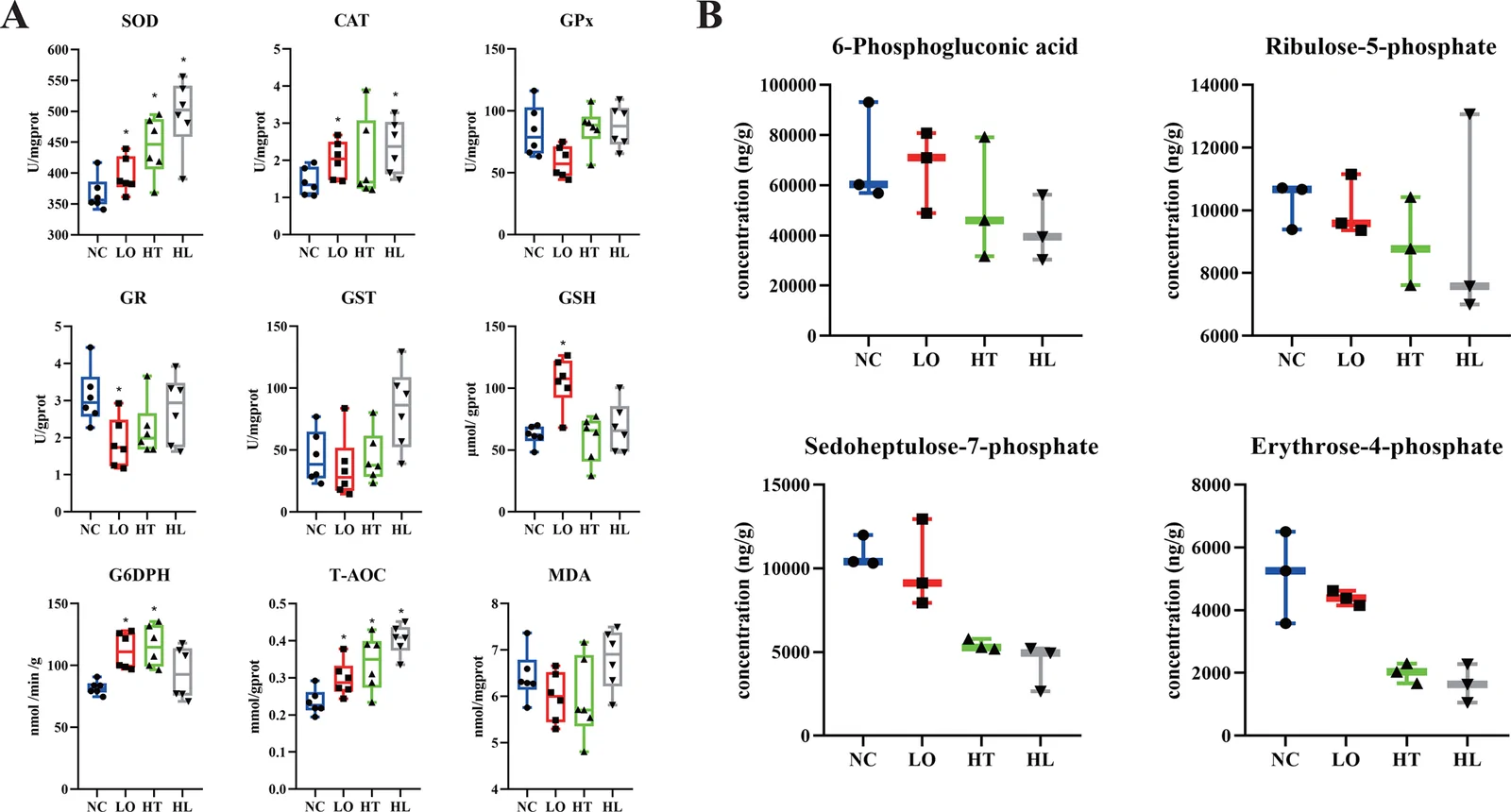
Effects of heat and hypoxia stress on enzyme activities, antioxidants and metabolites. * indicates *p* values below 0.05. (**A**) Enzyme activities and antioxidants. (**B**) Metabolites concentration of the pentose phosphate pathway

Under hypoxia stress, SOD and CAT activities, along with GSH and T-AOC levels significantly increased, whereas GR activities significantly decreased (*p* < 0.05). Under heat stress, SOD activity and T-AOC levels demonstrated a significant increase (*p* < 0.05). Furthermore, under combined heat and hypoxia stress, SOD and CAT activities, along with T-AOC levels, showed a significant increase (*p* < 0.05). Notably, the MDA level did not change significantly in the experimental groups. Additionally, the mean gill G6PDH activity in LO and HT demonstrated a significant increase compared to NC (*p* < 0.05) (Fig. 1A).

The concentration of sedoheptulose-7-phosphate and erythrose-4-phosphate were decreased in HT and HL groups than NC group, although the differences were not significant (Fig. 1B). No significant changes were found in the pentose phosphate pathway tested metabolites in the LO group (Fig. 1B).

**Fig. 2:**
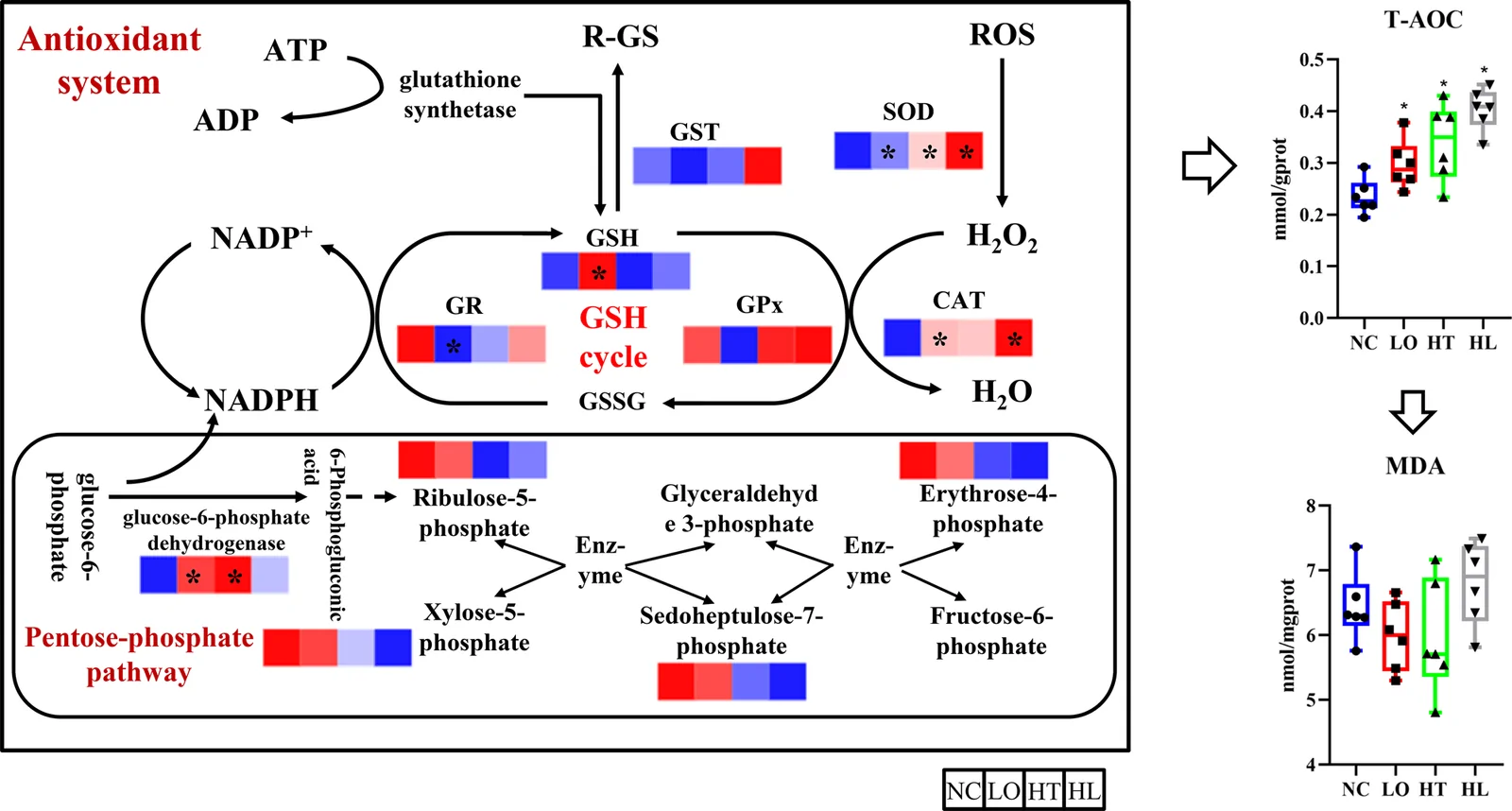
Systemic analysis of antioxidant response to environmental stress. Color indicates the values of metabolites from the highest (red) to the lowest (blue). * indicates *p* values below 0.05

Taken together, the hard clam can activate the antioxidant system, increase T-AOC capacity, and maintain relatively stable MDA level after stress. The antioxidant system was activated under heat and hypoxia stress showing stress-specific. GSH cycle might acted as the first line to protect antioxidant stress under hypoxic condition. SOD and CAT also played an important role in antioxidant protection under hypoxia stress. The pentose phosphate pathway might also took part in alleviating oxidative stress under heat and combined stress (Fig. 2).

### Widely targeted lipidomic profile

**Fig. 3:**
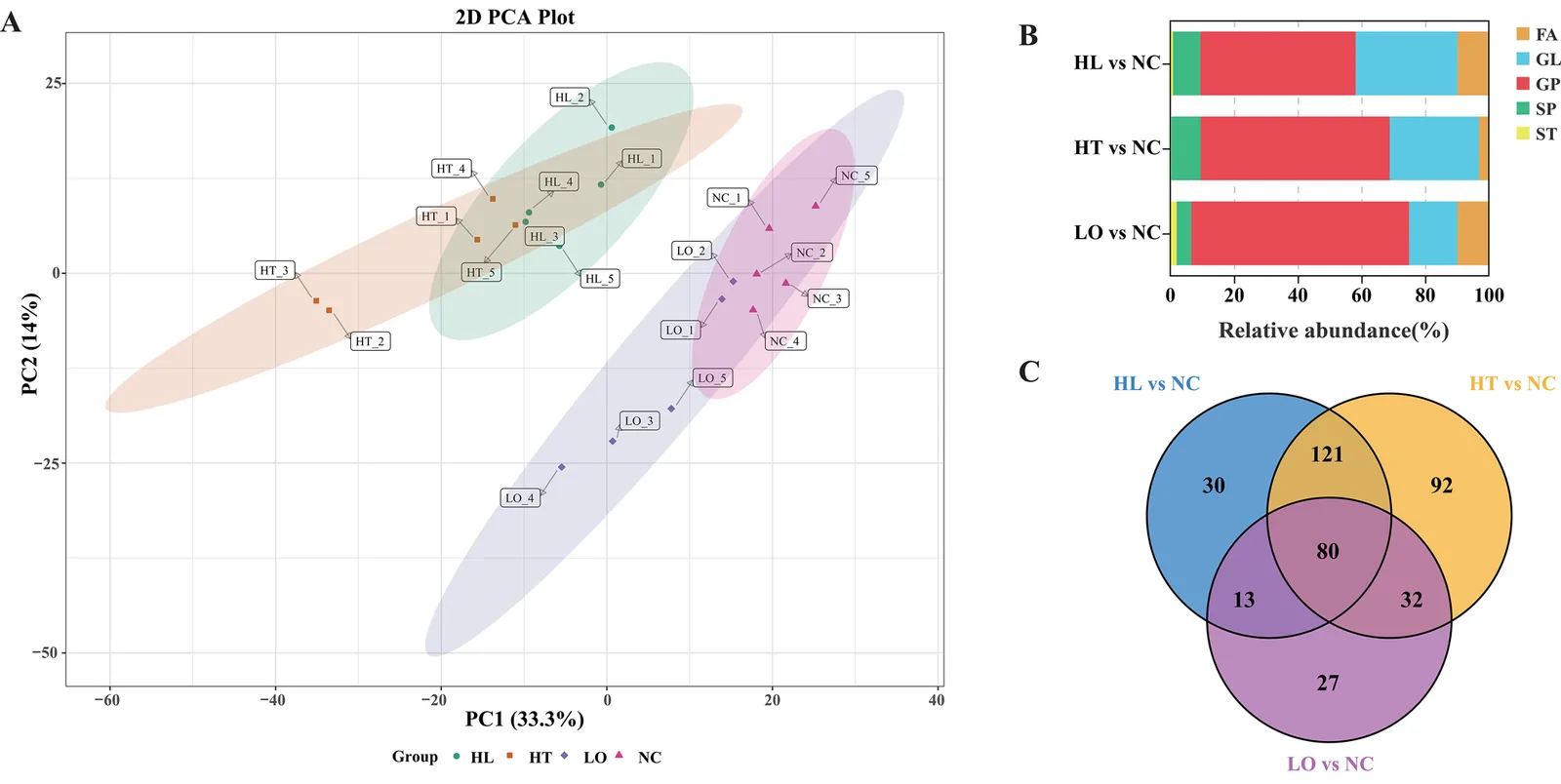
Lipidomic analysis under heat and hypoxia stress in the hard clam. HL, Heat and hypoxia group; HT, Heat group; LO, Hypoxia group; NC, control group. (**A**) Principal component analysis (PCA) of the lipid metabolites in gills. The ellipses represent a 95% confidence interval. (**B**) Composition and percentage of differential expression lipid metabolites under environmental stresses. (**C**) Venn diagram of differential expression lipid metabolites pairwise comparison

Due to maintain relatively stable MDA level after stress, the widely targeted lipidomic analysis was employed to explore the response mechanism at the lipid metabolite level. Overall, 913 lipid metabolites were identified based on the Metware self-built MWDB database. The PCA analysis based on the lipid metabolites revealed that high temperature groups (HL and HT) were different from the metabolites of hard clams in the normal temperature groups (LO and NC) on first principal component (PC1) accounting for 33.3% variability (Fig. 3A).

OPLS-DA analysis was employed to obtain more obvious separation. The results showed that three pairs of pairwise comparisons were readily separated and did not indicate any specific outliers in the samples (Fig. S1). The differential expression lipid metabolites were identified between stress groups (HL, HT and LO) and control group. The details of differential expression lipid metabolites number of each comparison were shown in Table S1. Based on LIPIDMAPS classification, the differential expression lipid metabolites mainly contained five classes, including Fatty acyl (FA), glycerolipids (GL), glycerophospholipids (GP), sterol lipids (ST), and sphingolipids (SP). The Fig. 3B showed the composition percentages of five classes of the lipid metabolites. Among these, GP showed the largest percentage (48.77% in HL vs NC, 59.38% in HT vs NC and 68.42% in LO vs NC). Although the percentage of differential GP metabolites of metabolome in HL vs NC, HT vs NC, and LO vs NC was 10%, 20.12%, and 0, respectively [14]. This may be because metabolome analysis mainly focuses on hydrophilic metabolites, which can be supplemented by further lipidomics studies [41]. Significant lipid changes were not restricted to GP; instead, they encompassed many major lipid classes, demonstrating the broad role of various lipids in the hard clam’s stress response. Moreover, GL also showed the high percentage (31.97% in HL vs NC, 28% in HT vs NC and 15.13% in LO vs NC). A total of 395 unique differential lipid metabolites were identified. 80 shared differential lipid metabolites including 1 ST, 1 FA, 6 SPs, 17 GLs, and 55 GPs (Fig. 3C).

**Fig. 4:**
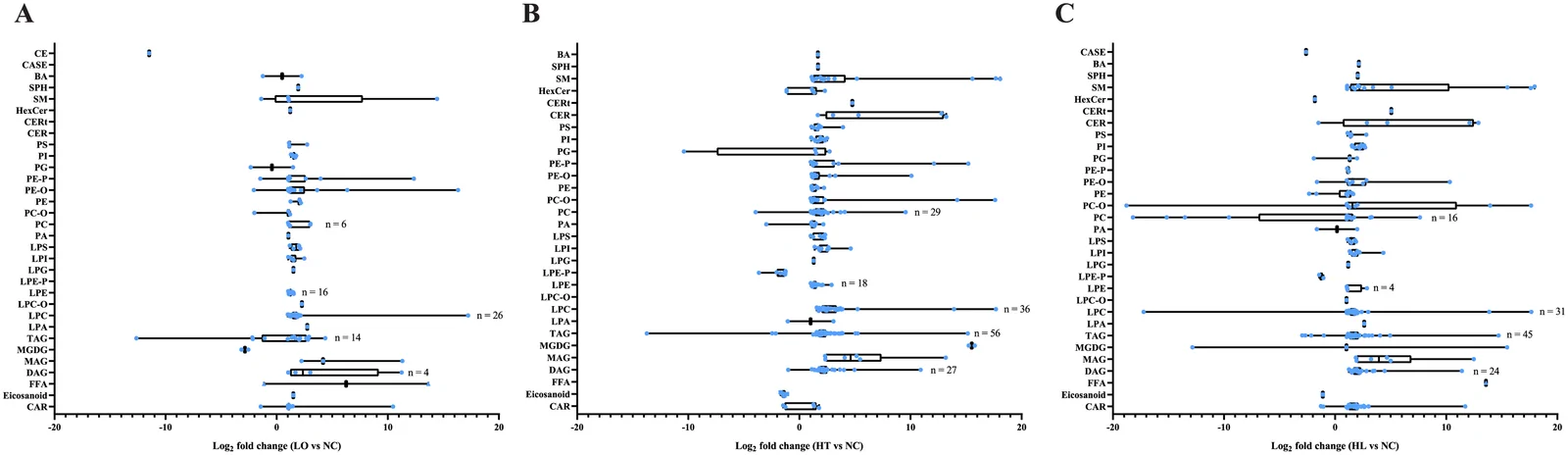
Variability of lipid class in response to hypoxia (**A**), heat (**B**) and combined stress (**C**). Each data point represents the value of log_2_(fold change) between stress group and control group

In the subclass of GP, Lysophosphatidylcholine (LPC) showed obviously differential altered under hypoxia, heat and combined stress (the numbers of differential expression lipid metabolites were 26, 36, 31, respectively). Lysophosphatidylethanolamines (LPE) showed obviously differential altered under hypoxia and heat stress (the numbers of differential expression lipid metabolites were 16 and 18, respectively). Phosphatidylcholine (PC), phosphatidylacid (PA), phosphatidylserine (PS) also showed obviously differential altered under heat stress (Fig. 4). In the subclass of GL, diglycerol (DAG) and triglycerol (TAG) showed obviously differential altered under heat and combined stress. While the numbers of differential expression DAG and TAG under hypoxia stress were obviously less than heat and combined stress. In the subclass of SP, ceramide (Cer) and sphingomyelin (SM) showed obviously differential altered under heat and combined stress (Fig. 4).

### KEGG enrichment analysis of differential expression lipid metabolites

**Fig. 5:**
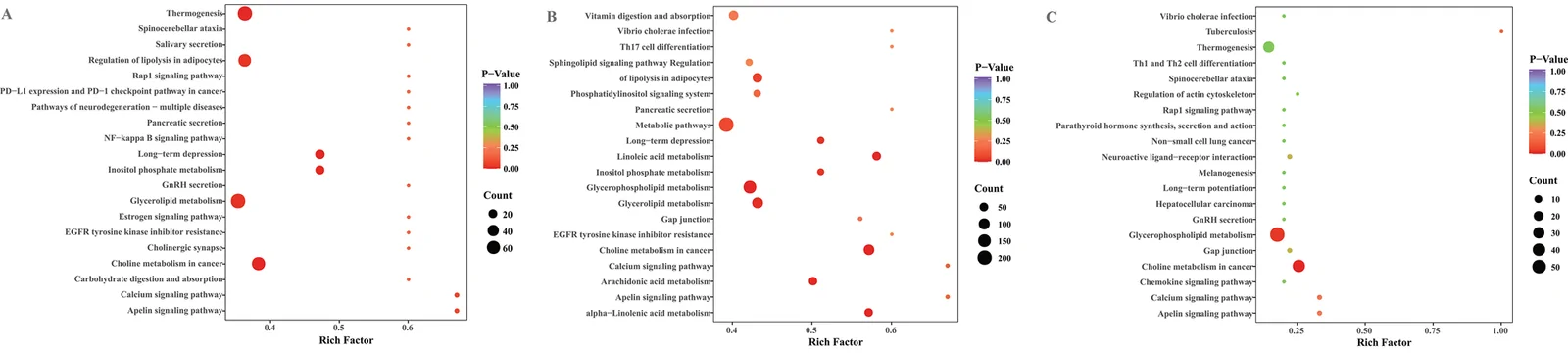
Enriched KEGG pathways for the differential expression lipid metabolites. (**A**) HL vs NC. (**B**) HT vs NC. (**C**) LO vs NC

Choline metabolism in cancer, Long-term depression, Thermogenesis, Inositol phosphate metabolism, Glycerolipid metabolism and Regulation of lipolysis in adipocytes were significantly enriched in HL vs NC (Fig. 5A). Choline metabolism in cancer, Linoleic acid metabolism, alpha-Linolenic acid metabolism, Glycerophospholipid metabolism, Arachidonic acid metabolism, Glycerolipid metabolism and other pathways were significantly enriched in HT vs NC (Fig. 5B). Choline metabolism in cancer and Glycerophospholipid metabolism were significantly enriched in LO vs NC (Fig. 5C).

### Expression analysis of lipid metabolism related genes

**Fig. 6:**
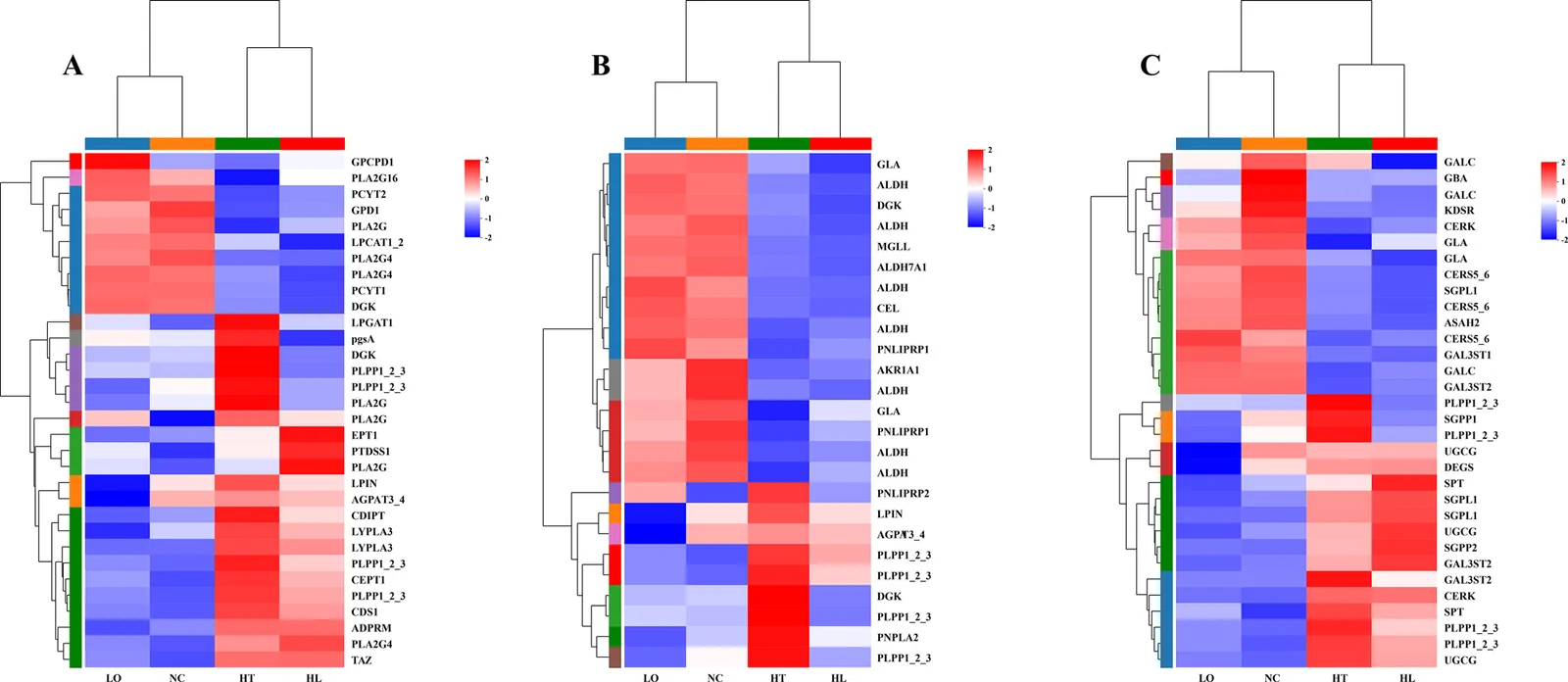
Heatmap of differentially expression genes in lipid metabolism pathways. (**A**) Glycerophospholipid metabolism (map00564). (**B**) Glycerolipid metabolism (map00561). (**C**) Sphingolipid metabolism (map00600)

To reveal the effects of heat and hypoxia stress on lipid metabolism, we further explored the transcriptome data and analyzed the expression patterns of lipid metabolism related genes. According to KEGG functional annotation and differential expression analysis, 231, 6 and 190 genes related to lipid metabolism were differentially expressed after heat, hypoxia and combined stress, respectively. Since GL, GP and SP were the main differential expression lipid metabolites, we investigated the differential expression genes involve in Glycerophospholipid metabolism (map00564), Glycerolipid metabolism (map00561) and Sphingolipid metabolism (map00600). In Glycerophospholipid metabolism, most of differential expression genes were upregulated under HL and HT stress (Fig. 6A). For example, one copy of *Phospholipase A2 group IV* (*PLA2G4*) showed highly expression in HL and HT group, while two copies of *PLA2G4* showed highly expression in LO and NC group. In Glycerolipid metabolism, four copies of *phospholipid phosphatase 1* (*PLPP1*) genes showed highly expression in HT group (Fig. 6B). In Sphingolipid metabolism, the *Ceramide* (*CER*) genes showed differential expression after stress (Fig. 6C).

### Systemic analysis of lipidomic response to environmental stress

**Fig. 7:**
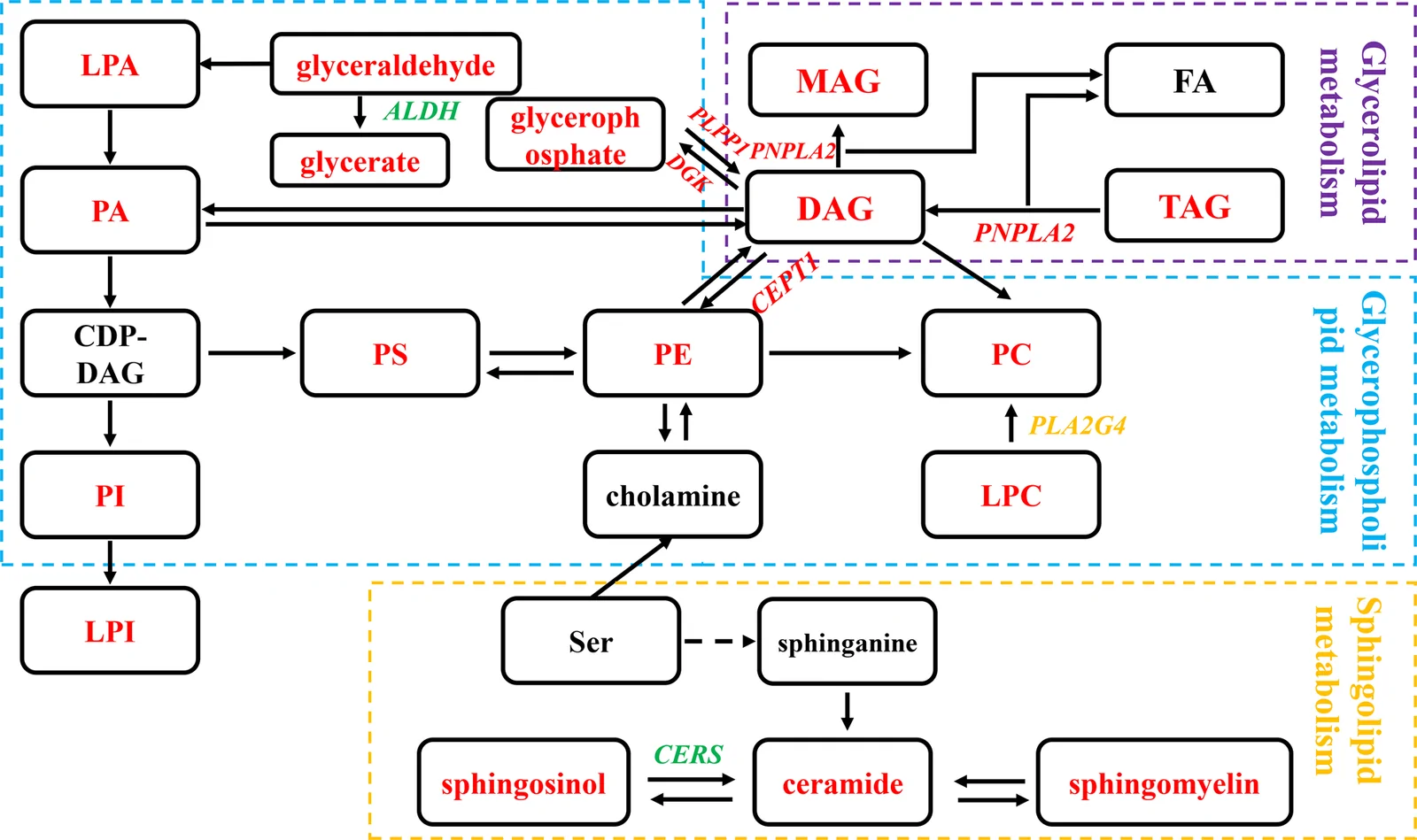
The hypothetical lipidomic response map under environmental stresses. Standardized form indicates lipid metabolite; Italic form indicates gene. Red indicates increase or upregulate; Green indicates decrease or downregulate; Orange indicates both increase or decrease

Potential lipidomic response mechanisms of hard clam to environmental stress were as shown in Fig. 7. There was a complex transformation between different classes of lipids. The lipid remodeling of the gill tissue was mainly caused by GP, GL and SP metabolism in the hard clam. In Glycerophospholipid metabolism, most of differential expression Glycerophospholipid were upregulated under stress. The expression of *ethanolamine phosphotransferase (CEPT1)* was significantly increased, which can convert DAG into Phosphatidylethanolamine (PE), resulting in increase of PE content. Lysophospholipids (LPs) can be converted into phospholipids, such as Lysophosphatidic acid (LPA) can be converted into PA, LPC can be converted into PC [41]. The increase of phospholipid content might be an adaptive response to stress. In Glycerolipid metabolism, the content of most differential expression glycerols (monoglycerol MAG, DAG and TAG) increased significantly after stress. Moreover, the significant increase of DAG content also provided raw materials for the synthesis of GP (such as PE, PC). In Sphingolipid metabolism, Cer, SM and sphingolipid (HexCer) were significantly accumulated under stress.

## Discussion

Oxidation imbalance in marine invertebrates is a common response induced by environmental stress [20, 53]. Overproduction of ROS could induce lipid peroxidation and damage the membrane system [20, 54]. Organism employs various strategies to cope with oxidative damage, including activating the antioxidant system [55]. Since lipids were the main components of the biological membrane and play a vital role in adaptation of organisms to environmental stress, lipidomics can provide insights into adaptive responses of organisms to a heat and hypoxic environment and help to illustrate potential novel roles of lipids in the process of heat and hypoxic adaption. Our previous study has shown that some small molecule antioxidants including ascorbic acid, myoinositol were differential altered under hypoxia, heat and combined stress [14]. Moreover, lipid metabolites account for nearly one-third of the total differential expression metabolites response to stress [14]. These results indicated that antioxidant response and fine-scale lipid remodeling might be more closely related to heat and hypoxia tolerance. In the present study, we explored the antioxidant pattern under hypoxia, heat and combined stress. Our results showed that the antioxidant system was enhanced showing stress-specific to increase T-AOC capacity and maintain relatively stable MDA level. In order to overcome the bias of metabolomics to hydrophilic metabolites [41], the widely targeted lipidomic analysis were employed to explore the lipid metabolism. Our results showed that lipid remodeling was mainly caused by GP, GL and SP metabolism.

The antioxidant system was activated under environmental stress showing stress-specific. Preparation for Oxidative Stress (POS) hypothesis implied that increased endogenous antioxidant protection occurred under situations that reduce oxygen availability, and prepared/protected animal tissues from an expected surge in oxyradical formation during reoxygenation as an anticipatory response [56, 57]. In the present study, the SOD and CAT activities, along with GSH content significantly increased, whereas GR activities significantly decreased under hypoxia stress. SOD and CAT are vital enzymes of defense against oxidative stress, through by catalyzing the dismutation of O^2 –^ into H_2_O_2_ [28] and disproportionation of H_2_O_2_ into H_2_O and O_2_ [29], respectively. The activities of SOD and CAT were significantly increased in the early duration of hypoxia stress to defense against oxidative stress in pearl oyster *Pinctada fucata martensii* (Dunker, 1880) [34]. SOD activity also enhanced in the gill of mussel *Mytilus edulis* (Linnaeus, 1758) under 0.6 mg/L hypoxia stress [58]. GSH is a kind of thiol-containing compounds serve as an important non-enzymatic antioxidant in the organism, and act as substrates for antioxidases [21, 59]. Due to adequate GSH could eliminate ROS, the elevated content of GSH could be a protective mechanism to prevent cells from oxidative stress. GSH-related enzymes are crucial in the antioxidant defense system [21]. GR and GPx involve in GSH recycle to play vital roles in antioxidant [21]. GR can reduce one molecular Oxidized glutathione (GSSG) to two molecular GSH [21]. GPx can catalyze the reduction of H_2_O_2_ or hydro-peroxides to water and reduce two molecular GSH to one molecular GSSG [60]. In the present study, the content of GSH significantly increased while the GR activity significantly decreased under hypoxia stress, these results indicated that GSH might act as vital antioxidant protector under hypoxia stress in the hard clam. After 15 days and 30 days of hypoxia stress, the level of GSH increased in experimental groups than control condition in mussel *Mytilus coruscus* (Gould, 1861) [25]. In another study, the GSH content shows a time-dependent decreased in both *Larimichthys crocea* (Richardson, 1846) liver and cell line under hypoxia stress, which was explained by GSH acts as a substrate for GPx and GR during antioxidation [54]. Either decrease or increase GSH content shows species, tissue and stress specific response.

Compared with normal temperature, elevated temperature can accelerate the enzymatic reaction to generate excessive ROS. Organism can employ antioxidant enzymatic reaction or non-enzymatic antioxidant to minimize cellular damage caused by ROS [60, 61]. For example, Manila clams *Ruditapes philippinarum* (Reeve, 1850) significantly increased SOD and CAT activities in response to heatwaves [35]. The pentose phosphate pathway (PPP), considered the branch from glycolysis, serves as a primary source of nicotinamide adenine dinucleotide phosphate (NADPH) generated from an oxidative branch and is required to synthesize ribonucleotides generated from a nonoxidative branch [62, 63]. Glucose-6-phosphate serves as the intersection point of glycolysis and PPP, where it undergoes dehydrogenation by G6PDH to produce NADPH [62, 63]. During the GSH cycle, GR utilizes NADPH to reduce one molecular GSSG to two molecular GSH [21]. Our present results showed that the activity of G6PDH was increased in LO and HT groups indicating that the PPP took part in redox maintain under environmental stress in hard clams. Moreover, other PPP intermediates showed obviously decreased in HT and HL groups. The PPP is tended to enhance the oxidative branch to provide more NADPH if cells are under stress condition for which maintaining redox homeostasis is more important than nucleic acid synthesis [62]. Perhaps the hard clams enhance the PPP oxidative branch to relieve ROS stress under heat and combined stress.

T-AOC is a universal indicator evaluating the total antioxidant capacity level in marine bivalves [25, 34, 64, 65]. It is an indicator to reflect the entire antioxidative system capacity to eliminate ROS [66]. Although the hard clam adopted different antioxidant strategies, our results showed that the T-AOC were significantly enhanced under heat, hypoxia and combined stress. MDA is a typical lipid peroxide biomarker. The MDA level did not change significantly in gill and mantle of scallops *Chlamys farreri* (Jones & Preston 1904) after heat stress [65]. Previous study showed that organic osmolytes (e.g., myoinositol) and vitamins (e.g., ascorbic acid) might help the hard clam relieve oxidative stress [14]. Combined with the present antioxidant enzyme and non-enzyme antioxidant results, the level of MDA did not significantly increase suggesting that lipid peroxidation was relieved.

In the present study, lipid remodeling was mainly caused by GP, GL and SP metabolism under hypoxia, heat and combined stress. Biological membrane system is sensitive to temperature, lipids (such as GPs and SPs) are important components of cell membrane. So, it should not ignore the lipid metabolism under stress. The temperature-responsive remodeling of membrane lipids, known as homeoviscous adaptation [67], which can change in the relative proportions of membrane lipids, such as phospholipids and sphingolipids [68, 69].

GPs can be divided into PC, PE, PS, PA, etc. based on polar head group [41]. Especially for PC and PE, are major building blocks of membrane bilayers. Heat stress can significantly affect the GP metabolism [70–72]. For example, a large amount of GP metabolites changed significantly under heat stress in *Scophthalmus maximus* (Linnaeus, 1758), *Paralichthys olivaceus* (Temminck & Schlegel, 1846) and *Oncorhynchus mykiss* (Walbaum, 1792) [70, 72, 73]. The content of LPC showed significantly increased following 6 hours of hypoxic stress, and the contents of PC, LPC and PE were also showed significantly increased following 24 hours of hypoxic stress in sea cucumber *Apostichopus japonicus* (Selenka, 1867) [74]. Our results showed that GPs showed the largest proportion of all differential expression lipid metabolites, which was similar with the results of *Salvelinus alpinus* (Linnaeus, 1758) [69]. In addition, the content of PC, PA, and PS showed obviously differential altered under heat stress. Moreover, LPC showed obviously differential altered under hypoxia, heat and combined stress. The elevated levels of PA and PS in warm tolerance *C. angulata* compared to cold tolerance *C. gigas* [39]. PC followed by LPC are the main heat adapted lipid molecules, highlighting the vital role in short term heat adaptation [39]. However, the content of most LPE and LPC species were significantly decreased response to elevated temperature in *S. alpinus* [69]. The inconsistent of lysophosphatide variation trend between vertebrate and invertebrate might be caused by distinct environment and evolutionary pressures, and reflected species-specific membrane adaptations [39].

SPs mainly divide into Cer, SM and HexCer based on structures [75]. Cer enhances mitochondrial membrane permeability [76]. SM is also a basic component of cell membranes. Although the expression of *ceramide synthase* gene was significantly decreased in HT and HL groups, the Cer and SM showed obviously differential altered under heat and combined stress. Previous studies showed that *Carassius carassius* (Linnaeus, 1758) adapted to warmer temperatures are more abundant in SM [77], and the abundance of most SMs increased significantly after warmer exposure in *S. alpinus* [69]. Our results showed that the content of most sphingomyelin increased significantly after stress. Wang et al. (2021) noted that accumulation of sphingomyelin can counteract the fluidization effect caused by heat stress and help to stabilize the membrane structure [69]. Taken together, the increase content of most GP and SP metabolites to change in the relative proportions of membrane lipids was vital to stabilize the membrane structure under hypoxia and heat stress in the hard clam.

The accumulation of intracellular protective agents to alleviate ROS damage is an adaptive strategy. TAG is the main storage form of lipid energy metabolism, and also serve as cache of fatty acids for membrane lipids remodeling [69]. Complete hydrolysis of TAG can produce fatty acids and glycerol. DAG is a key intermediate of lipid metabolism and serve as a key precursor of de novo biosynthesis of lipids [69, 78]. DAG can be hydrolyzed to produce fatty acids and glycerol. And it can also produce PE and PC under the action of CEPT1 and EPT1, respectively. In the present study, TAG and DAG showed obviously differential altered under heat and combined stress, while the numbers of differential DAG and TAG under hypoxia stress were obviously less than heat and combined stress. The upregulation of *Patatin Like Domain 2, Triacylglycerol Lipase (PNPLA2)* was conducive to the hydrolysis of glycerides. Previous studies showed that the TG content was significantly increased in *Corythucha ciliata* (Say, 1832) [79] and *S. alpinus* [69], suggesting that their heat protectant roles. Besides, the accumulation of diglycerol provided raw materials for the synthesis of GPs (such as PE, PC).

## Conclusion

In the present study, coupled lipidomic and biochemical approaches were employed to reveal the response mechanism to heat and hypoxia stress at the antioxidant stress and lipid metabolism level in the hard clam. Activation of the antioxidant system which evidenced by elevated T-AOC, can alleviate oxidative damage, thereby preventing excessive accumulation of MDA under environmental stress. Concurrently, increased abundances of GP and SP metabolites drive remodeling of membrane lipids, which is critical for maintaining membrane fluidity and structural integrity during stress. Furthermore, the accumulation of GL metabolites functions as protective agent. This study expands our understanding of hard clam responses to heat and hypoxia stress at the antioxidant and lipid metabolism level, and provide new insights into the stress adaptation mechanism in marine organisms. Future studies should aim to identify key lipid metabolites associated with adaptive potential and elucidate their underlying biological functions.

## Electronic supplementary material

Below is the link to the electronic supplementary material.

Supplementary Material 1

Supplementary Material 2

## Data Availability

The lipidomic, enzyme and targeted metabolomic data in the present study are accessible from the supplementary materials. The RNA-seq raw data was deposited in the NCBI SRA (PRJNA973806).
