# Supplementary material for: Response mechanism to heat and hypoxia stress in hard clam: insights from antioxidation and lipid metabolism
Source: Front Zool. 2026 Jun 30;23:32. doi: 10.1186/s12983-026-00622-5 (PMC13591755; doi:10.1186/s12983-026-00622-5)
Supplement: Supplementary file 1 [file 12983_2026_622_MOESM2_ESM.docx]

**Supplementary Materials**

**Response mechanism to heat and hypoxia stress in hard clam: insights from lipid metabolism and antioxidation**

Zhi Hu ^a, b, c, 1^, Mei-Jie Yang ^b, c, d, 1^, Xing-Hao Ma ^e^, Pu Shi ^b, c, d^, Tao Zhang ^b, c, d,^ *, Hao Song ^b, c, d, *^

**a** Key Laboratory of Mariculture of Ministry of Education, Ocean University of China, Qingdao 266003, P. R. China

**b** CAS Key Laboratory of Marine Ecology and Environmental Sciences, Institute of Oceanology, Chinese Academy of Sciences, Qingdao 266071, China

**c** Center for Ocean Mega-Science, Chinese Academy of Sciences, Qingdao 266071, China

**d** University of Chinese Academy of Sciences, Beijing 100049, China

**e** Shouguang Marine Fishery Development Center, Weifang 262700, China

* Corresponding authors: Tao Zhang (tzhang@qdio.ac.cn), Hong Song (haosong@qdio.ac.cn)

Institute of Oceanology, Chinese Academy of Sciences, 7 Nanhai Road, Qingdao, Shandong 266071, China.

Tel/fax number: +86-532-82898705;

1 Zhi Hu and Mei-Jie Yang contributed to the work equally.

**1. The details of UPLC** **analytical conditions:**

(1) column: Thermo Accucore™ C30 (2.6 μm, 2.1 mm*100 mm i.d.);

(2) solvent system A: acetonitrile/water (60/40, V/V, 0.1% formic acid, 10 mmol/L ammonium formate),

solvent system B: acetonitrile/isopropanol (10/90 V/V, 0.1% formic acid, 10 mmol/L ammonium formate);

(3) gradient program: A/B 80:20, V/V at 0 min, 70:30 V/V at 2.0 min, 40:60 V/V at 4 min, 15:85 V/V at 9 min, 10:90 V/V at 14 min, 5:95 V/V at 15.5 min, 5:95 V/V at 17.3 min, 80:20 V/V at 17.3 min, 80:20 V/V at 20 min;

(4) flow rate: 0.35 ml/min;

(5) temperature: 45 °C;

(6) Injection volume: 2μl.

**2. The details of MS conditions:**

The ESI source operation parameters:

(1) ion source: turbo spray;

(2) source temperature: 500 °C;

(3) ion spray voltage (IS): 5500 V (Positive), -4500 V (Neagtive);

(4) ion source gas 1 (GS1): 45 psi, gas 2 (GS2): 55 psi, curtain gas (CUR): 35 psi;

(5) the collision gas (CAD): medium.

**3. The details of targeted metabolite analysis conditions:**

(1) column: ACQUITY UPLC BEH Amide;

(2) solvent system A: water with 10mM Ammonium acetate and 0.3% Ammonium hydroxide, solvent system B: 90% acetonitrile/water;

(3) gradient program: 95% B (0-1.2 min), then decreased to 70% B (8 min), 50% B (9-11 min), and finally ramped back to 95% B (11.1-15 min).

**4. RNA-seq** **libraries construction**

RNA was isolated using a TRIzol kit (Invitrogen, CA, USA). RNA quality was evaluated via agarose gel electrophoresis (111860, Biowest, Spain). RNA concentration, as well as OD260/280 and OD260/230 ratios, were quantified using a ND-2000 spectrophotometer (NanoDrop Technologies). The RNA integrity number was determined using the RNA 6000 Nano Kit (5067-1511, Agilent, USA) on a Bioanalyzer 2100 system (Agilent, USA). Library preparation for sequencing was performed with the NEBNext® Ultra™ RNA Library Prep Kit (Illumina, NEB, USA). Cluster generation was conducted using a cBot Cluster Generation System, with the TruSeq PE Cluster Kit v3-cBot-HS (Illumina, NEB, USA) for indexing.

**Table S1** The numbers of differential lipid metabolites by pairwise comparison

| Pairwise comparison | Up | Down |
| --- | --- | --- |
| LO vs NC | 137 | 15 |
| HT vs NC | 301 | 24 |
| HL vs NC | 220 | 24 |

(A)

(B)

(C)

**Fig.S1** The score plots of OPLS-DA pairwise comparisons. (A) HL vs NC. (B) HT vs NC. (C) LO vs NC.
